# Study of the Catalytic Strengthening of a Vacuum Carburized Layer on Alloy Steel by Rare Earth Pre-Implantation

**DOI:** 10.3390/ma12203420

**Published:** 2019-10-18

**Authors:** Guolu Li, Caiyun Li, Zhiguo Xing, Haidou Wang, Yanfei Huang, Weiling Guo, Haipeng Liu

**Affiliations:** 1 School of Materials Science and Engineering, Hebei University of Technology, Tianjin 300130, China; liguolu@hebut.edu.cn (G.L.); licaiyun0301@163.com (C.L.); 2 National Key Laboratory for Remanufacturing, Academy of Army Armored Forces, Beijing 100072, China; huangyanfei123@126.com (Y.H.); guoweiling_426@163.com (W.G.); 3 Process Institute of Inner Mongolia First Machinery Group Co, Ltd, Baotou Inner Mongolia 014030, China; sheb008@163.com

**Keywords:** 20Cr2Ni4A, vacuum carburizing, ion implantation, rare earths, catalysis, carbon diffusion, microstructure

## Abstract

Conventional carburizing has disadvantages, such as high energy consumption, large deformation of parts, and an imperfect structure of the carburizing layer. Hence, a rare earth ion pre-implantation method was used to catalyze and strengthen the carburized layer of 20Cr2Ni4A alloy steel. In this study, X-ray diffraction (XRD), X-ray photoelectron spectroscopy (XPS), optical microscopy (OM), scanning electron microscopy (SEM), energy dispersive microanalysis (EDS), transmission electron microscopy (TEM), and Rockwell/Vickers hardness testing were used to analyze the microstructure, phase composition, retained austenite content, hardness, carburized layer thickness, and carbon diffusion. The results showed that lanthanum and yttrium ions implanted into the 20Cr2Ni4A steel formed solid solutions of rare earth ions and a large number of dislocations, which improved the diffusion coefficient of carbon elements on the carburized surface and the uniformity of the carbon distribution. Simultaneously, rare earth ion implantation improved the structure and hardness of the vacuum carburized layer. Compared to the lanthanum ion implantation, yttrium ion implantation caused the structure of the carburized layer to be finer, and the carbon diffusion coefficient increased by 1.17 times; in addition, the surface hardness of the carburized layer was 61.8 HRC.

## 1. Introduction

Since 20Cr2Ni4A alloy steel has excellent surface properties after carburization, it has been widely used for gears and bearings in heavy-duty equipment that is frequently exposed to harsh conditions, such as heavy loads, elevated working temperatures, high frequency vibrations, and high rotational speeds [1,2]. Hence, failures such as wear, pitting, and contact fatigue damage, are more likely to occur on the surface of gears and bearings [3,4,5]. Vacuum carburization, as a surface strengthening method, plays an important role in the process of obtaining excellent comprehensive mechanical properties of 20Cr2Ni4A alloy steel. Vacuum carburization is a rapid process in which acetylene is injected into a high-temperature furnace in pulsed mode under low pressure and vacuum [6,7,8]. However, vacuum carburizing still has its disadvantages, such as a high carburizing temperature, a long carburizing cycle, an uneven structure, and coarse carbide particles. Studies showed that the introduction of rare earth (RE) ions in the carburizing heat treatment of steel or other parts reduces the treatment temperature and increases the carbon activity and the carbon diffusion coefficient during the carburization process; it can also improve the structure of the carburized layer [9,10]. Yan et al. [11,12] studied the effect of an RE catalyst on the gas carburizing kinetics of steel and found that compared to the conventional gas carburizing process, the incorporation of an RE led to an obvious increase in layer depth and carbon concentration profile, under the same carburizing conditions. The diffusion coefficient and transfer coefficient increased by 50% and 117%, respectively. Dong et al. [13] used RE ion implantation to assist vacuum carburizing of 12Cr2Ni4A steels. The results showed that the addition of RE elements obviously improved the hardness of the carburized layer and decreased the hardness gradient. Moreover, the structure of the carburized layer was compact, and the carbides were fine and dispersed.

Ion implantation is a modern material surface modification technology that has become increasingly common for changing the characteristics and properties of metal materials [14]. It can selectively improve the wear resistance, corrosion resistance, oxidation resistance, and fatigue resistance of the material surface by implanting a small amount of metal ions without changing the original properties of the material substrate [15,16,17]. In recent years, ion implantation of lanthanum and yttrium has been mainly focused on enhancing the corrosion resistance, high-temperature oxidation resistance, and biocompatibility of pure metals or alloys [18,19,20]. Xu [21] discovered a significant improvement in the aqueous corrosion resistance of zircaloy-4 by yttrium ion implantation, compared to that of the as-received zircaloy-4. Wang [22] obtained the same conclusion upon exploring the lanthanum ion implantation into a zirconium alloy. Darwin et al. [23] found that ion implantation of lanthanum significantly improved the high-temperature oxidation resistance of an Fe80Cr20 alloy and developed a promising interconnection metal material for planar-type solid oxide fuel cells, namely, Fe80Cr20 60h-La. The co-implantation of yttrium and carbon ions enhanced the wear resistance of H13 steel, which was shown by an increase in the microhardness of 60%–170% and a wear resistance of 2–3 times [24].

However, there have been only a few studies on the application of REs for vacuum carburizing of alloy steel, and the detailed structure and surface properties of the carburizing layer have seldom been analyzed. Thus, to meet the surface property requirements of 20Cr2Ni4A alloy carburized steel, ion-implanted lanthanum and yttrium were used to improve the hardness and surface properties. Due to the large radius of REs, substantial lattice damage and high-density structural defects are produced in the alloy steel matrix during the process of ion implantation. After carburizing, the surface analysis was studied in detail by X-ray photoelectron spectroscopy (XPS), transmission electron microscopy (TEM), X-ray diffraction (XRD), scanning electron microscopy (SEM), and optical microscopy (OM). In addition, the Rockwell and Vickers hardness tests were used to evaluate the hardness of the carburized layer on three different treated samples. Lastly, the catalysis mechanism and the strengthening mechanism of REs during carburization are discussed.

## 2. Materials and Methods

The parent material of the 20Cr2Ni4A alloy steel (0.17 wt% C, 0.25 wt% Si, 0.45 wt% Mn, 1.45 wt% Cr, 3.48 wt% Ni, and 0.01 wt% Mo) was cut into samples with dimensions of 15 mm × 15 mm × 15 mm. Before ion implantation, these samples were polished with a series of silicon carbide (SiC) abrasive papers with grits of 600# to 2000#, polished with a W3.5 diamond paste and finally ultrasonically cleaned with anhydrous alcohol, for 10 min. Lanthanum and yttrium with 99.99% purity were implanted with a metal vapor vacuum arc (MEVVA) ion source (Shanghai Kingstone Semiconductor Joint Stock Limited Company, Shanghai, China) at the Institute of Low Energy Nuclear Physics in Beijing Normal University (China). The parameters of the ion implantation process are shown in Table 1. Vacuum carburization was carried out after ion implantation of the REs. Vacuum carburization was carried out for 5 hours in a vacuum environment (1 × 10^−5^ Pa) with a carbon potential of 1.2% and a temperature of 920 °C. To promote the uniform diffusion of carbon atoms, C_2_H_2_ and N_2_ were injected alternately for 25 pulse cycles. Five hours after carburizing at 920 °C, high temperature tempering at 650 °C for 3 hours promoted the precipitation of carbides and the decomposition of retained austenite; then oil quenching at 820 °C was done to improve the hardness and toughness of the material; finally, low temperature tempering was carried out at 180 °C for 3 hours, to eliminate the internal stress of the sample. The whole heat treatment process was completed on the vacuum carburization automatic production line (ECM Technologies, Grenoble, France).

The chemical characterization of the implanted surface and the depth profile of the element content distribution of the samples, with depth, were performed by XPS (Thermo Scientific Escalab EI+) (Thermo Fisher Scientific, Waltham, MA, America) using a monochromatic Al radiation source (energy = 55 eV). The acceleration voltage of argon ion etching was 4 kV, and the rate was 5 nm/min. The binding energy of elements was normalized to 284.8 eV of carbon. To illustrate the crystal structure defects of the implantation area after ion implantation, the surfaces of the implanted samples were observed using TEM (JEM-2010) on an instrument equipped with energy dispersive spectroscopy (EDS) (JEOL, Tokyo, Japan). The TEM samples for surface observation by ion implantation were prepared according to the following procedure. Thinning samples were bonded to silicon wafers using epoxy resin glue. Then, the sample were sliced vertically along the plane of the implanted surface and machined to a thickness of about 10 μm. Finally, the samples were ground by Gatan 691 ion thinner (Gatan, Pleasanton, CA, USA) at a small incident angle between 8°and 3°.

**Figure 1:**
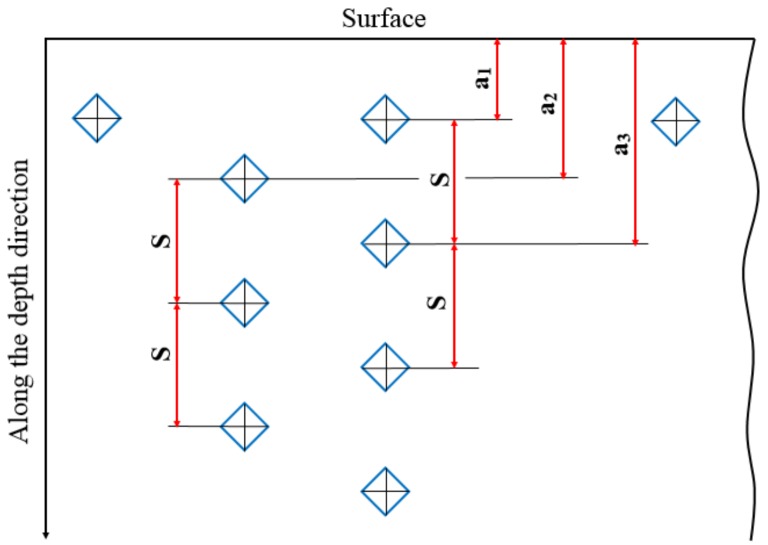
Locations of the hardness indentations.

The phase composition was studied by XRD (D8 Advance, Bruker AXS, Karlsruhe, Germany, Cu Kα1 = 1.5406 Å) at 40 kV and 40 mA, with an incident angle of 0.5°, after ion implantation and vacuum carburizing. The scanning speed and angle range were 1°/min and 10°–90°, respectively. The penetration depths of X-ray was estimated to be 26 nm at incident angles of 0.5° [25]. Considering the maximum concentration distribution depth of the two implanted ions at 20–25 nm, 0.5° was chosen as the incident angle of the XRD test after carburization. The content of the retained austenite was determined with an X-350A X-ray stress analyzer (ST Stress Technology Co., Ltd., Handan, China). The surface morphology and phase structure of the samples were observed by Optical Microscope (OM) (Olympus, Tokyo, Japan) and a Nova NanoSEM50 environmental SEM (FEI, Hillsboro, OR, USA) equipped with EDS (OXFORD, Oxford, England) was also used. The corrosive fluid for OM comprised an alcohol solution containing 4% nitric acid (volume fraction). To obtain the effective case depth of the carburized layer, according to the GB/T 9450-2005 standard, Vickers hardness testing was used to determine the longitudinal section hardness of the samples under a 1 kgf (HV_1_) load, and each hardness value was the average of three measurements. In the Vickers hardness testing, the distance (S) between two adjacent indentation centers should not be less than 2.5 times of the indentation diagonal and the distance difference between successive adjacent indentation centers and part surfaces (a_2_–a_1_,) should not exceed 0.1 mm, as shown in Figure 1. The load time of the apparatus was 15 seconds. The Rockwell hardness of the surface and the core of the samples was measured by a Rockwell hardness tester (Shjingmi, Shanghai, China), and each hardness value was the average of five measurements. For the carburized surface, the depth of Rockwell indenter was about 0.08 mm; for the center, the depth of the Rockwell indenter was about 0.14 mm. The carbon element distribution on the surface of the carburized layer was determined by an electron probe microanalyzer (EPMA-1720, Shimadzu, kyoto, Japan). The test parameters were as follows—acceleration voltage, 10 kV; electron beam current, 200 nA; beam spot, 20 um; and test time, 20s.

## 3. Results

### 3.1. Calculation of the Implanted Ion Range

**Figure 2:**
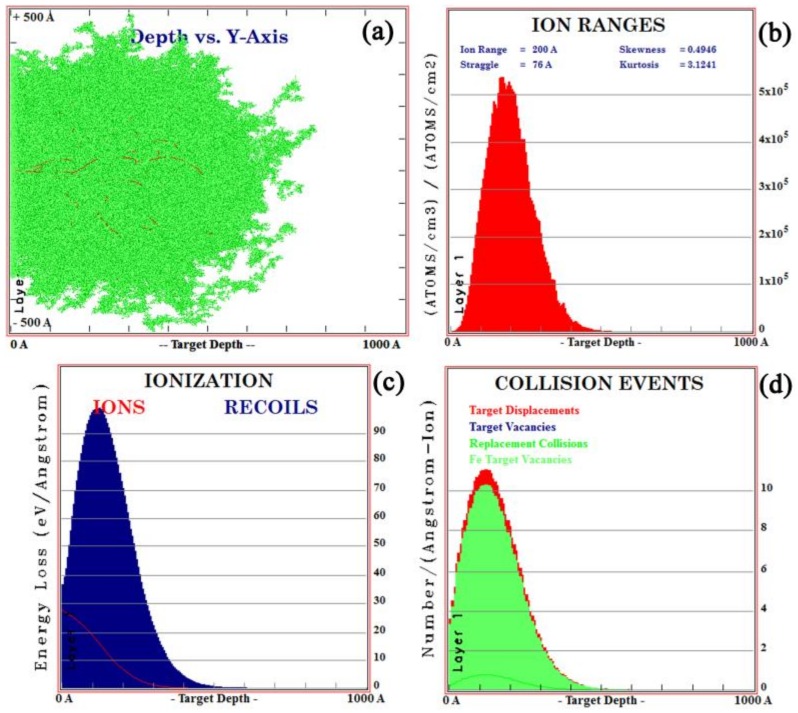
TRIM simulation of lanthanum ion implantation for the (**a**) ion beam pattern, (**b**) ion ranges, (**c**) ionization distribution of ions, and (**d**) collision events.

**Figure 3:**
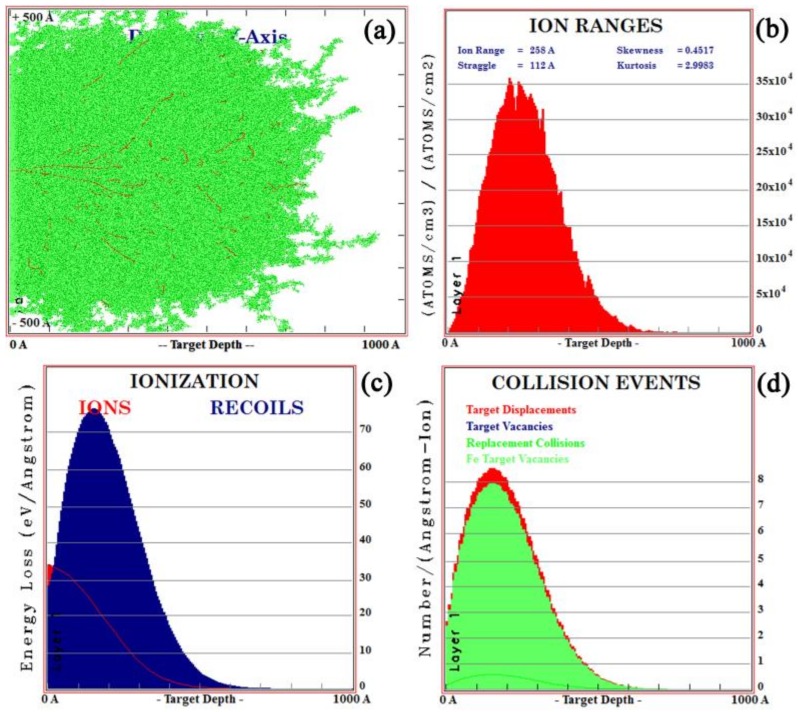
TRIM simulation of yttrium ion implantation for the (**a**) ion beam pattern, (**b**) ion ranges, (**c**) ionization distribution of ions, and (**d**) collision events.

The SRIM is commonly used to simulate the ion sputtering process [26]. In this case, the ion range of all implanted ions in the simulation was calculated using dynamic simulation (TRIM) [27]. The impurity distributions of lanthanum and yttrium ions at ionic energies of 100 Kev and 105 Kev were simulated by TRIM. The results are shown in Figure 2 and Figure 3, respectively. As shown in Figure 2a and Figure 3a, each time an implanted ion collided with a target atom, a vacancy (lines of red dots) would be created, which would cause cascade damage (clusters of green dots) of the target atoms in steel. As shown in Figure 2b and Figure 3b, the maximum ionic ranges of lanthanum and yttrium were 50 and 60 nm, respectively, and both had a normal distribution, which was consistent with the XPS results. Figure 2c and Figure 3c show the ionization distribution in which lanthanum and yttrium ions lose their energy in steel samples, and it was observed that the host lattice arrangement of steel was damaged during the implantation of lanthanum and yttrium ions (Figure 2d and Figure 3d).

### 3.2. Chemical Composition and Structure of the Implanted Surface Layer

**Figure 4:**
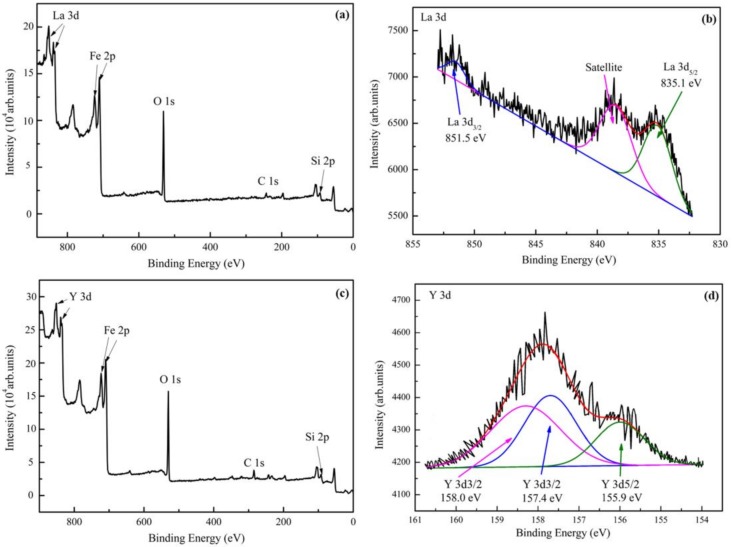
XPS spectra of sample surfaces—(**a**) survey spectrum and (**b**) La 3d spectrum of implanted La; (**c**) survey spectrum and (**d**) Y 3d spectrum of implanted Y.

Figure 4 shows the XPS spectra of the samples after La and Y ion implantation, where contaminants on the surface were removed by Ar^+^ etching for 1 minute. Figure 4a,c show the full XPS spectra, which indicate that there are iron, oxygen, carbon, and silicon on the implanted lanthanum and yttrium surfaces, respectively. Compared with the binding energy of the standard absorbed carbon of 284.8 eV, the surface energy of the absorbed carbon in this study was 285.2 eV, which was 0.4 eV higher than that of 284.8 eV. The adjusted XPS data of La and Y are separately illustrated by Figure 4b,d respectively. Figure 4b shows the La 3d surface XPS spectrum. The three peaks correspond to La 3d_3/2_ (851.5 eV) and La 3d_5/2_ (835.1 eV). The spin-orbit splitting value was 16.4 eV, which clearly suggest a typical La_2_O_3_ pattern [28]. The phenomenon was also observed by Jin et al. [29]. Figure 4d shows the Y 3d XPS spectrum, and the peaks at 158.0 eV and 155.9 eV correspond to the Y–Y bond, and the peak at 157.4 eV corresponds to Y_2_O_3_. Consequently, this result illustrate that lanthanum mainly exists as oxides, while yttrium exists as oxides and metallic Y in the RE implanted layer.

**Figure 5:**
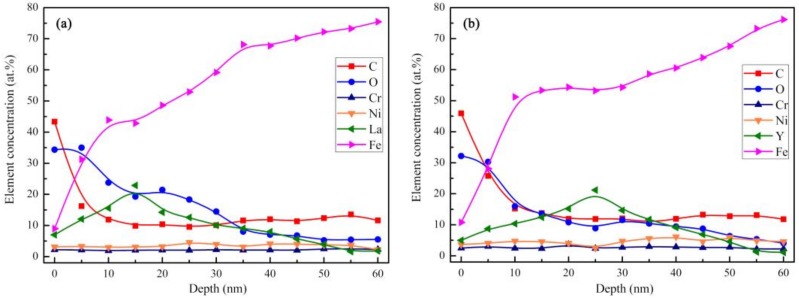
Depth profiles of element content distribution obtained by XPS—(**a**) lanthanum implantation result and (**b**) yttrium implantation result.

Figure 5 shows the depth profiles of the element content distribution of lanthanum and yttrium ion implantation samples from the XPS tests. Figure 5a,b show that the concentration of lanthanum and yttrium in the ion implantation layer present a normal distribution trend, and the peak concentration distributions are at depths of approximately 15 and 25 nm, with concentrations of 22.9 wt% and 21.2 wt%, respectively.

**Figure 6:**
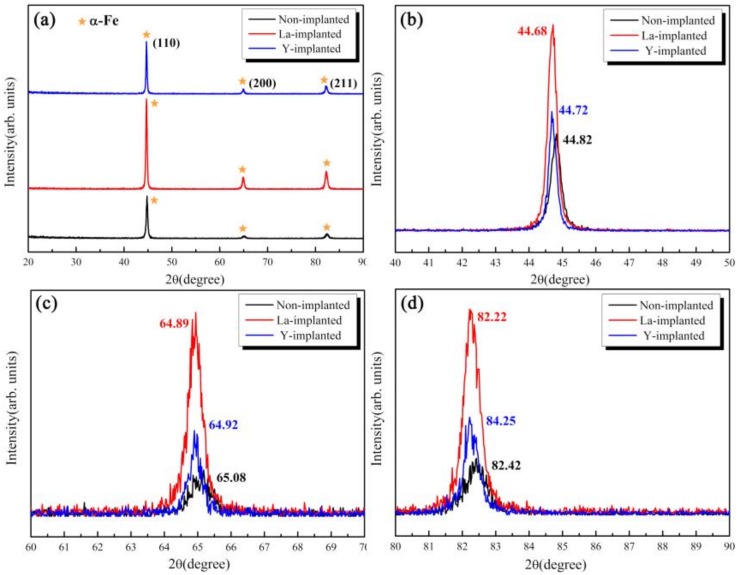
XRD patterns of the implanted surface with and without rare earth (RE) implantation—(**a**) XRD spectra; (**b**) diffraction angle of the (110) peak; (**c**) diffraction angle of the (200) peak; and (**d**) diffraction angle of the (211) peak.

As shown in Figure 6, due to the larger radius of rare earth and the internal stress introduced by ion implantation [30], all three diffraction peaks of α-Fe shift to the left and the peak intensity increased, which meant that the lattice distortion occurred on the surface of the implanted matrix, according to the Bragg equation [31].

**Figure 7:**
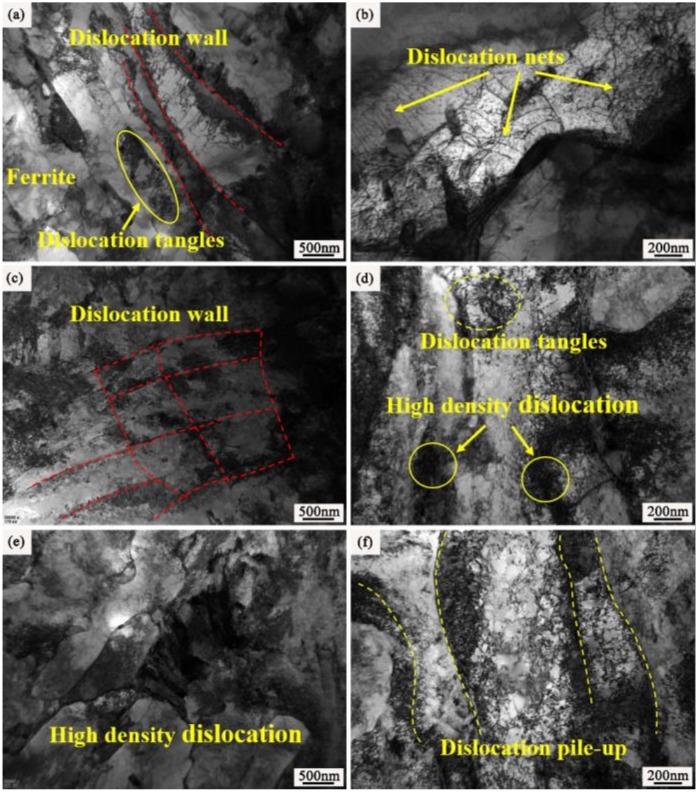
TEM observation of non-carburized samples after ion implantation—(**a**,**b**) non-implanted sample; (**c**,**d**) lanthanum-implanted sample; and (**e**,**f**) yttrium-implanted sample.

Figure 7 shows the TEM image of the surface layer of the samples, with and without ion implantation. It can be observed from Figure 7a,b that that there are some crystal defects in the 20Cr2Ni4A matrix, such as dislocation entanglement and dislocation grid, which form a stable defect network. The RE ion implantation caused lattice damage to the body-centered cubic (BCC) Fe matrix. From the observation of Figure 7c–f, the matrix implanted with rare earth ions forms high density dislocation entanglement compared to that of the non-implanted sample. Especially after yttrium ion implantation, the matrix was almost full of high-density dislocations, which accumulated and tangled [30].

### 3.3. Phase and Microstructure of the Carburized Layer After Ion Implantation

**Figure 8:**
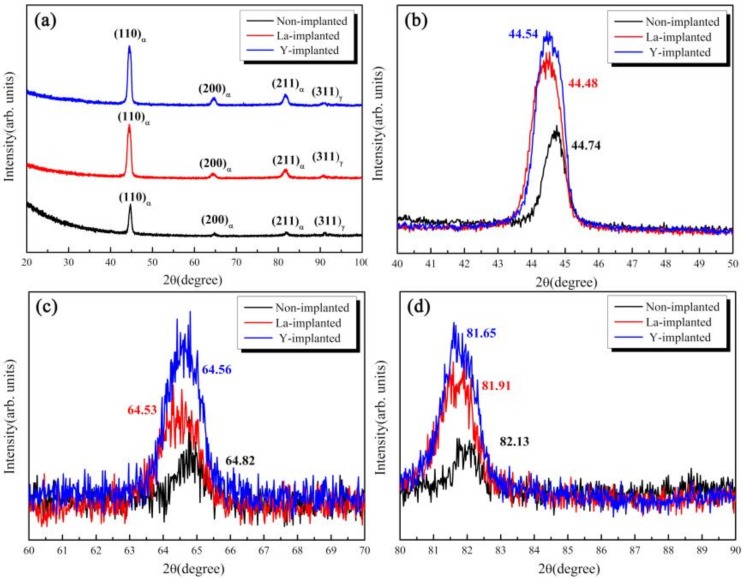
XRD spectra of the carburized layers of the three samples—(**a**) XRD spectra; (**b**) diffraction angle of the (110) peak; (**c**) diffraction angle of the (200) peak; and (**d**) diffraction angle of the (211) peak.

The phase on the surface of the carburized samples was measured by XRD, as shown in Figure 8. The carburized layers of the three samples are composed of a martensite phase with a body-centered cubic (BCC) structure and retained austenite with a face-centered cubic (FCC) structure. Due to the limitation of the phase detection spatial resolution with XRD, there are no diffraction peaks from lanthanum and yttrium. However, it was found that the strongest diffraction peaks from the lanthanum and yttrium-implanted samples shift to the left by 0.26 and 0.20 degrees, respectively, compared with that of the non-implanted sample. The other two peaks of martensite migrated to the left at the same time. In addition, the intensity of all crystal planes after lanthanum and yttrium implantation were stronger than those from the non-implanted sample. X-ray stress analyses also revealed that the content of the retained austenite in the carburized layer decreased slightly with the implantation of RE ions, as shown in Table 2.

**Figure 9:**
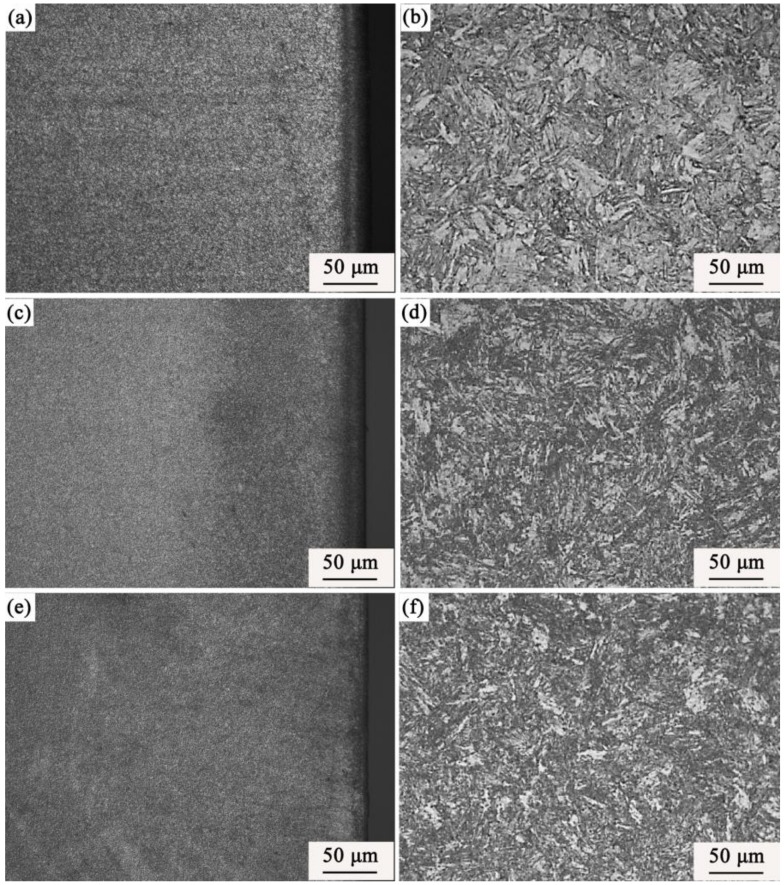
Microstructure of the carburized layer on the three samples—(**a**) carburized surface layer without RE; (**b**) core after carburizing without RE; (**c**) carburized surface layer after La implantation; (**d**) core after La implantation; (**e**) carburized surface layer after Y implantation; and (**f**) core after Y implantation.

In this section, the microstructures of the carburized layers of different samples carburized in vacuum for 5 hours at 920 °C are discussed. Figure 9a,c,e show the layers from conventional carburizing and carburizing with REs lanthanum and yttrium, respectively. Figure 9a shows that the microstructure of the carburized layer was composed of a martensite matrix, and granular carbides were dispersed in it and retained austenite, which were not transformed into martensite. However, ultrafine acicular martensite and fine dispersed grain carbides were obtained in the carburized layer after the ion implantation of lanthanum and yttrium, as shown in Figure 9c,e, respectively. Figure 9b,d,f show that the core microstructures of the three samples were tempered martensite and free ferrite. According to the GB/T 25744-2010 metallographic standard, the core microstructures of the non-implanted sample were equivalent to grade 4, while those of the samples with RE ion implantation were equivalent to grade 3.

**Figure 10:**
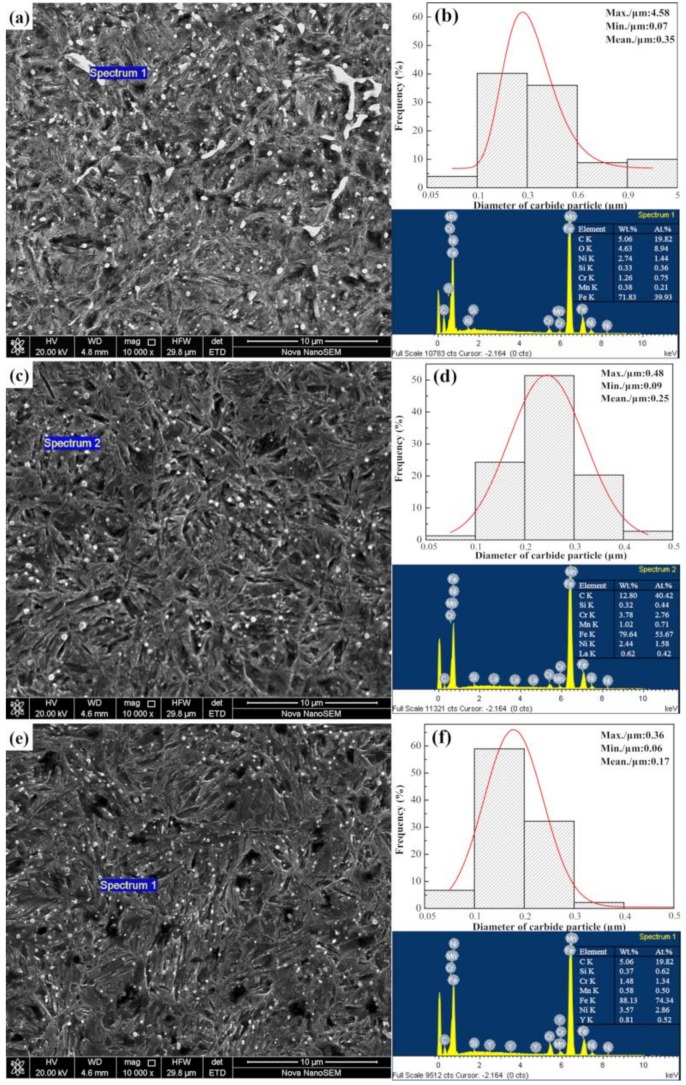
Diameter of the granular carbides in the carburized surface layer—(**a**) carbide morphology and energy dispersive spectroscopy (EDS) results without RE and (**b**) its carbide diameter distribution; (**c**) carbide morphology and EDS results with La implantation and (**d**) its carbide diameter distribution; (**e**) carbide morphology and EDS results with Y implantation, and (**f**) its carbide diameter distribution.

In addition, the SEM images of the carbides in the carburized layer and their corresponding size and distribution histograms are shown in Figure 10. Figure 10a shows that the majority of the carbides are granular and unevenly distributed in the interstices between the martensites and are accompanied by large diameter bar carbides. However, compared to that in the non-implanted layer, the carbide distribution in the carburized layer of the sample after ion implantation was finer and more uniform. Among the non-implanted and implanted layers, the carbides implanted with yttrium were the finest. The EDS measurements of the carbide particles in the three samples showed that the carbides were mainly composed of carbon, iron, chromium, nickel, and manganese, while lanthanum and yttrium were found in the carbides in the ion-implanted samples. The size distribution of the carbide particles was estimated by using Nano Measurer 1.2 software and Gaussian fitting (v1.2, Wan An Intelligent Technology, Wuhan, China). At least 150 particles were counted from each SEM image. The carbide particles in SEM photos were labeled and statistical reports were derived [32]. The average diameter of carbides on the surface of the non-implanted samples was 0.35 μm. Among them, 81.2% of the carbides were within 0.6 μm in diameter, and the length of a portion of the large strip carbides was more than 5 μm. However, the average diameters of the carbides on the surface of the carburized layer after ion implantation of lanthanum and yttrium were 0.25 and 0.17 μm, respectively. The diameters of most carbides were from 0.1 to 0.3 μm. The results showed that ion implantation of the REs reduced the particle size of carbides on the carburized layer surface. The effect of yttrium ion implantation was better than that of the lanthanum ion implantation because yttrium implantation resulted in a minimum particle size of 0.06 μm and a maximum particle size of 0.36 μm. In other words, RE ion implantation pretreatment played an important role in refining the structure of the carburized layer and promoting the dispersion of the fine carbide precipitates.

### 3.4. Hardness Distribution and Effective Hardening Depth of the Carburized Layer

**Figure 11:**
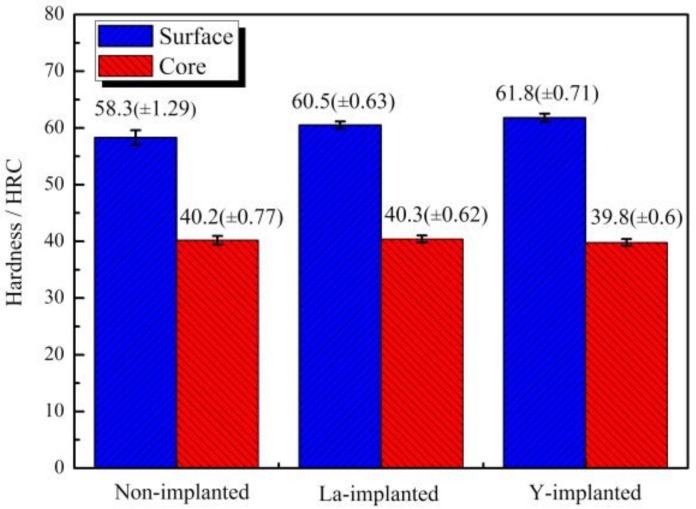
Rockwell hardness of the carburized layer and the core of the three samples.

Figure 11 shows the results of the Rockwell hardness measurements. The surface Rockwell hardness of the non-implanted sample was 58.3 HRC, and the hardness of RE-implanted samples was higher than the previous value, especially samples with the yttrium addition. After vacuum carburization, the Rockwell hardness of the yttrium-implanted sample increased to 61.8 HRC at the subsurface layer; this value was higher than that of the lanthanum-implanted sample (60.5 HRC). In contrast, the core hardness value did not change significantly. The smaller error bar value indicated that there was no significant difference in the range of surface hardness.

**Figure 12:**
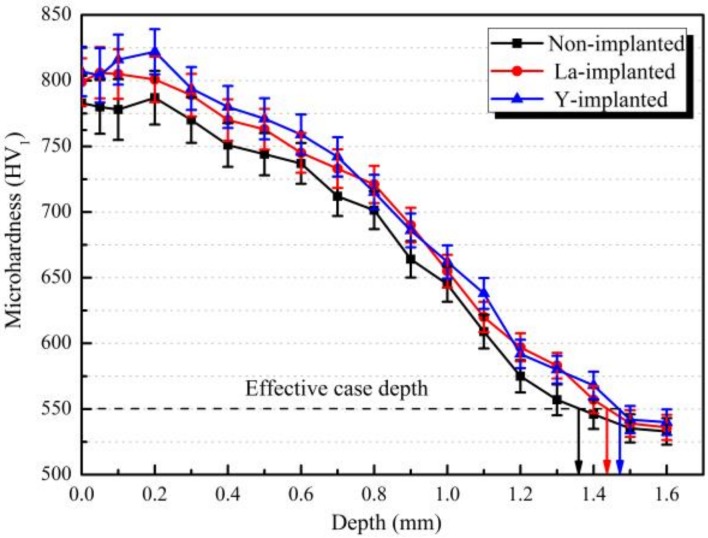
The microhardness distribution of the carburized layer along the depth direction.

Figure 12 shows the microhardness results of the samples after carburization, with and without the RE ions. The fluctuation of the error bars were not more than 25 HV_1_. It could be seen that the microhardness of the carburized layer after RE ion implantation was higher than that treated by conventional vacuum carburization at 920 °C, and the change in the hardness gradient was minor. The maximum hardness of the carburized layer after ion implantation of lanthanum and yttrium occurred at 0.1 mm and 0.2 mm from the surface and was 805 HV_1_ and 822 HV_1_, respectively, which was higher than the value of 778 HV_1_ at 0.2 mm from the surface, after conventional carburizing. Osman Asi suggested that the enhancement in hardness was mainly caused by the diffusion of carbon atoms [33]. According to the GBT 9450-2005 standard, the effective hardening depths of the non-implanted, La-implanted, and Y-implanted samples were 1.36 mm, 1.44 mm, and 1.47 mm, respectively, as shown by the arrows in Figure 12. RE ion implantation increased the hardness of the carburized layer of the 20Cr2Ni4A steel but did not cause a significant increase in the depth of the effective hardened layer. Hence, the results showed that the microhardness of the carburized layer obtained by vacuum carburizing with yttrium was the highest, and the change was the most uniform among the samples considered herein.

### 3.5. Calculation of the Carbon Diffusion Coefficient

**Figure 13:**
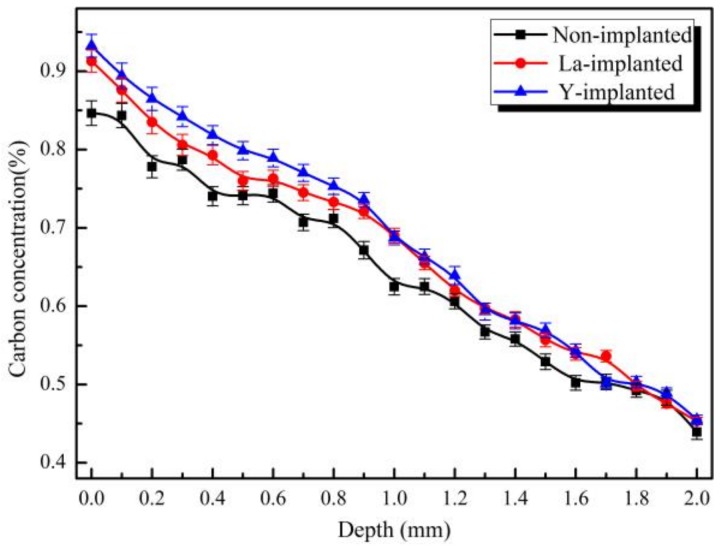
Carbon concentration along the depth of the carburized layers.

As can be seen from Figure 13, the carbon concentration on the carburized surface without ion implantation was about 0.84%, and the carbon concentration varied unevenly. After ion implantation, the carbon content on the carburized surface reached more than 0.9%, and the carbon concentration gradient changed more gently. The hardness gradient distribution of the three carburized specimens was approximately the same as that of the carbon element distribution measured by the EPMA.

During the vacuum carburization process, acetylene gas was the carbon source. The diffusion of carbon atoms followed Fick’s second law and was considered a one-dimensional plane diffusion phenomenon. If the diffusion coefficient did not change with the carbon concentration (or average diffusion coefficient), the carbon concentration distribution in the infiltration process satisfied Equation (1) [12]:

(1) $$\frac{\partial c\left(x,t\right)}{\partial_{t}}=D\frac{\partial^{2}c\left(x,t\right)}{\partial x^{2}}$$

where *c*(*x*, *t*) is the volume concentration of carbon, *x* is the distance from any point in the sample to the surface of the sample, *t* is the diffusion time, and *D* is the carbon diffusion coefficient.

With the extension of the carburizing time, the carbon concentration *c*_s_ on the steel surface gradually increased from the original carbon content *c*_0_ to a constant level and was in equilibrium with the carbon potential *c*_p_ of the furnace gas, which indicated that it had entered the stage of carbon diffusion. At the beginning of the carburization process, the initial and boundary conditions of Equation (1) were marked as *c*_0_ = (*x*, 0) and *c*_s_ = (0, *t*). The solution of Fick’s second law, which provided the curve of the carbon diffusion concentration, was as Equation (2):

(2) $$\frac{c_{x}-c_{0}}{c_{s}-c_{0}}=1-erf\left(x/2\sqrt{Dt}\right)$$

where *erf* (*x*/2$\sqrt{Dt}$) is the error function. When the specified value of *c_x_* was higher than *c*_0.35%_, the obtained depth of the hardened layer could be described as Equation (3):

(3) $$x=2K\sqrt{Dt}$$

where *K* is a constant (constant carbon potential).

Therefore, based on the depth of the carburized layer shown in Figure 12, when the carburizing temperature and time were the same, the diffusion coefficient relation of the carburized layer with or without RE ions could be obtained by Equation (3), as shown in Equations (4) and (5):

(4) $$1.44/1.36=\sqrt{D_{920{}^{\circ}C}^{La}/D_{920{}^{\circ}C}}$$

(5) $$1.47/1.36=\sqrt{D_{920{}^{\circ}C}^{Y}/D_{920{}^{\circ}C}}$$

where $D_{920{}^{\circ}}$, $D_{920{}^{\circ}}^{\mathrm{La}}$, and $D_{920{}^{\circ}}^{Y}$ are the carbon diffusion coefficients of 20Cr2Ni4A steel without ion implantation and with lanthanum or yttrium implantation before vacuum carburizing, respectively. Finally, the relations were shown in Equations (6) and (7):

(6) $$D_{920{}^{\circ}C}^{La}/1.12D_{920{}^{\circ}C}$$

(7) $$D_{920{}^{\circ}C}^{Y}/1.17D_{920{}^{\circ}C}$$

In short, the carbon diffusion coefficients after lanthanum and yttrium implantation were 1.12 and 1.17 times higher, respectively, than those of the non-implanted samples when the vacuum carburizing temperature was 920 °C and the carbon potential was 1.2%. This was consistent with the results of the hardness gradient distribution and the carbon concentration distribution of the carburized layer.

## 4. Discussion

### 4.1. Effect of RE on Carbon Diffusion

As shown in Figure 4 and Figure 5, after ion implantation, the 20Cr2Ni4A matrix formed an injection layer containing the RE, in which the RE atoms existed in the form of oxides and solid solution atoms. The results of the TEM analysis in Figure 7 showed that RE ion implantation caused substantial damage to the matrix lattice.

**Figure 14:**
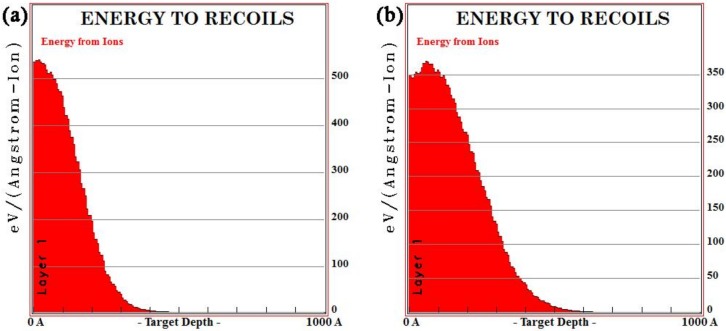
SRIM simulation of mean ion energy during ion implantation of (**a**) La and **(b**) Y into steel.

**Figure 15:**
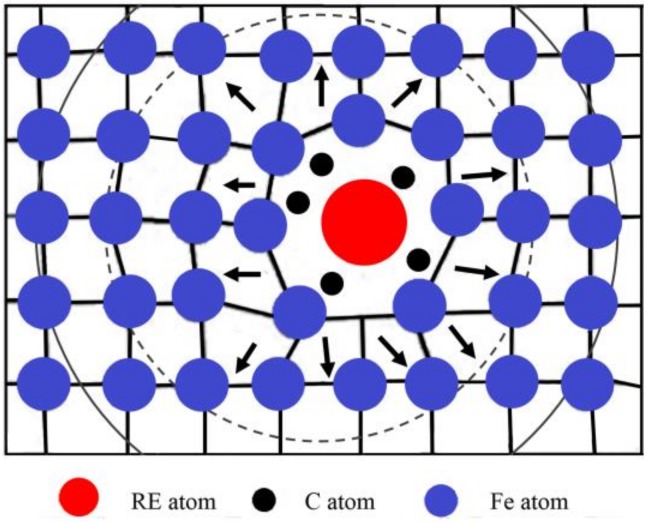
Physical model of an RE solid solution atom in a crystal face in an Fe lattice.

Due to the characteristics of the electron cloud of RE element shells and its atomic size effect, a series of cascade collisions occurred between ion-implanted RE atoms and substrate atoms, resulting in a large number of dislocations and damage in the matrix lattice [34,35]. The estimation of mean ion energy transferred to the target were reasonably evaluated by SRIM. The average primary knock-on atom energy of the La and Y ions were 94.6 and 96.8 Kev/ion during the ion implantation, respectively. As shown in Figure 14, the maximum electronic stopping powers of La and Y ions along the depth direction were 537.8 and 370.3 Kev/Å, respectively (electron stopping power is the blocking effect of nucleus and electrons in the target matrix on the implanted ions during ion implantation [34]). A schematic diagram is shown in Figure 15. Subsequently, the disorder degree of the matrix atoms, the crystal interface area and the crystal structure defects increased, which became the channels for carbon diffusion during carburizing and played an extremely important role in improving the carbon diffusion coefficient [11].

**Figure 16:**
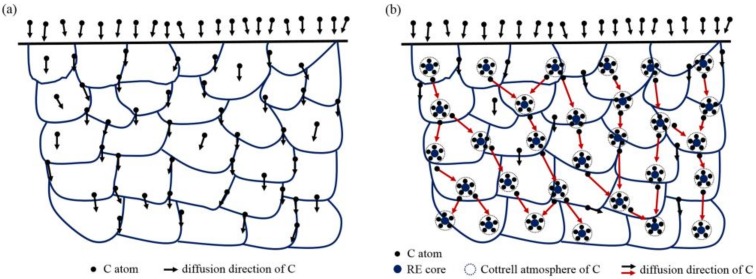
Diffusion models of (**a**) conventional carburizing and the (**b**) RE carburizing processes.

When solid solution RE atoms expand the lattice defects in the matrix, the distorted lattice region of the surrounding iron atoms becomes a trap for carbon atoms during carburization. Carbon atoms segregate into the voids of the distortion region [36], resulting in the formation of a nanoscale Cottrell atmosphere with the RE as the core carbon atom [2], as shown in Figure 16. Moreover, as shown in Figure 16a, carbon uniformly diffuses in the volume from the outside to the inside, in accordance with the concentration gradient in the austenite during conventional carburizing. The grain boundary diffusion was faster than that in the grains, and the finer the grain was, the faster was the diffusion rate. However, once the Cottrell atmosphere was formed during carburizing with RE ions, the diffusion mode changed from uniform diffusion to non-uniform diffusion [37]. As shown in Figure 16b, the Cottrell atmosphere also acted as an accelerator for diffusion [38]. First, the concentration of carbon atoms in the air mass was much higher than that in the matrix, resulting in a high concentration difference. Then, the larger the density of the air mass and the higher the average carbon concentration, the larger was the diffusion flux, such that the diffusion velocity and diffusion flux were significantly increased. Finally, the mechanism of carbon diffusion during RE carburization could be considered to be a result of jumping the short-circuit diffusion when the carbon atoms at the top of the last gas mass jumped to the next one. It could be concluded that the lattice damage and associated Cottrell atmosphere produced by the RE ion implantation changed the diffusion mode of the carbon atoms during the conventional carburizing processes and increased the diffusion coefficient of the carbon atoms.

### 4.2. Effect of REs on the Microstructure and Hardness of the Carburized Layer

According to the differences in the microstructure, phase, and hardness of the carburized layers between the non-implanted and implanted samples, as determined by the OM, SEM, and the XRD, it could be concluded that RE ion implantation can improve the carburized layer [10]. From Figure 8, the diffraction peaks of the ion-implanted lanthanum and yttrium specimens shift to the left, and the intensity of the peaks increased after vacuum carburizing. From the Bragg equation, the smaller the diffraction angle, the larger the lattice plane distance. Due to the large radius of RE atoms, the iron lattice in steel is distorted [39]. Moreover, the (110) α diffraction peak showed and obvious increase, which indicated that the preferred orientation of cryptocrystalline martensite was (110) α after RE ion implantation, and its content also increased. From the results in Figure 9 and Figure 10, it could be concluded that the structure of the carburized layer after ion implantation was obviously refined, the content of the cryptocrystalline martensite was increased, and the minimum size of the surface carbide was 0.17 μm. Figure 11 shows that the surface hardness increased after RE ion implantation.

**Figure 17:**
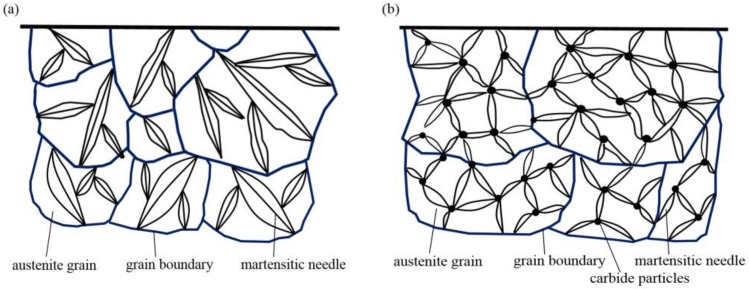
Transformation models of martensite in the grain interior for (**a**) conventional carburization and the (**b**) RE carburization processes.

These results could be explained according to the following two aspects. First, the RE ion implantation pretreatment could change the martensite transformation mode. Since martensite has an explosive shear growth, the first martensite could not pass through the austenite large-angle grain boundary, and it was impossible to cut through the carbides. During the conventional carburization process, substantial amounts of carbides do not exist in the austenite crystal, so the martensite tends to be coarse. Carbides precipitated from austenite crystals are often required during RE carburizing. In the presence of these carbides, the martensite shear is blocked and the martensite is forced to become superfine [10]. Moreover, the quenching performance was also improved. Figure 17 shows the martensite transformation patterns of the two carburizing methods. Figure 17a shows the transformation mode of austenite to martensite during conventional carburizing and quenching, and it can be seen that plate martensite needles often appeared. Figure 17b shows that during the carburizing and quenching processes with REs, the Cottrell atmosphere with RE ions as the core becomes the nucleation core for a carbide, which results in the precipitation of fine dispersed carbides on the surface, during carburization and makes the martensite superfine, as shown in Figure 10c,e. The fine, dispersed, spherical carbides were embedded in the martensite matrix, which inhibited the growth of the austenite grains and improved the hardness and wear resistance of the steel.

In addition, RE ion implantation pretreatment could dramatically refine the microstructure of the carburized layer. The undercooled austenite before quenching was uniform and stable during conventional carburization. RE carburization results in a typical non-uniform structure, as its stability is very poor due to the carbide nucleus [40]. Without undercooling, the carbon concentration around the carbide increased to the highest level and decreased far away from the carbide. The carbon concentration periodically changed according to the distance between the carbides. With an increase in the undercooling before quenching, the carbon saturation in austenite around the carbide increased sharply, causing the carbon atoms in the austenite to remain around the carbide. With the uphill diffusion process from the austenite to the carbide, that is, from a low concentration to a high concentration of carbon, the carbon in the austenite became depleted. After this transformation, the superfine martensite was formed. The resistance of the superfine martensite to fatigue crack initiation and propagation was greatly improved, so the microstructure contributed to increasing the service life of the different parts.

## 5. Conclusions

1. La and Y ion implantation can result in the appearance of La_2_O_3_, Y_2_O_3_ and Y (0). In the implanted layer, the maximum concentration of La and Y occurred at 15 and 20 nm, respectively, and the concentration of both ions showed a normal distribution.
2. During the carburization process, the high density of dislocation defects and the Cottrell atmosphere that formed on the surface of the steel by the RE ion implantation became the diffusion channels for the carbon atoms. During the carburization process at 920 °C and with a 1.2% carbon potential, the La ion implantation increased and the carbon diffusion coefficient was 1.12 times higher than that of the non-implanted steel, which was beneficial. Y ion implantation increased the carbon diffusion coefficient, which was 1.17 times higher than that of the non-implanted steel, which was also beneficial. The effective hardening depth of the carburized layer increased by 0.08 mm and 0.11 mm after the La and Y ion implantation, respectively.
3. RE ion implantation pretreatment could change the transformation mode of the martensite and could significantly refine the microstructure of the carburized layer. Most of the fine dispersed carbides have diameters ranging from 0.1 mm to 0.2 mm and the maximum diameters were less than 0.32 mm. The maximum diameter and minimum diameter of the carbides on the surface of the carburized layer after Y ion implantation were 0.36 mm and 0.06 mm, respectively. The surface hardness of the carburized layer after ion implantation of lanthanum and yttrium was 60.5 HRC and 61.8 HRC, respectively. The yttrium-implanted carburized layer had a higher surface hardness than the non-implanted carburized layer, showing an increase of 3.5 HRC. Therefore, the results showed that compared to lanthanum, yttrium has a better strengthening effect.

## Figures and Tables

**Table 1:** Parameters of ion implantation before carburization.

Sample	Implanted with La	Implanted with Y
Vacuum level (Pa)	3.5 × 10^−3^	3.5 × 10^−3^
Ion energy (Kev)	100	105
Implantation dose	2 × 10^17^ ions/cm^2^	2 × 10^17^ ions/cm^2^
Temperature (°C)	Room temperature	Room temperature
Element	La	Y

**Table 2:** Retained austenite content in the carburized surface layer of the three samples by XRD.

Sample	Non-Implanted	Lanthanum-Implanted	Yttrium-Implanted
Retained austenite	16.7%	15.6%	14.2%
